# Characterization of Extracellular Vesicles Labelled with a Lipophilic Dye Using Fluorescence Nanoparticle Tracking Analysis

**DOI:** 10.3390/membranes11100779

**Published:** 2021-10-12

**Authors:** Getnet Midekessa, Kasun Godakumara, Keerthie Dissanayake, Mohammad Mehedi Hasan, Qurat Ul Ain Reshi, Toonika Rinken, Alireza Fazeli

**Affiliations:** 1 Institute of Veterinary Medicine and Animal Sciences, Estonian University of Life Sciences, Kreutzwaldi 62, 51006 Tartu, Estonia; getnet.balcha@ut.ee (G.M.); kasun.godagedara@ut.ee (K.G.); keerthie.dissanayake@ut.ee (K.D.); mehedi.hasan@ut.ee (M.M.H.); qurat.reshi@ut.ee (Q.U.A.R.); 2 Department of Pathophysiology, Institute of Biomedicine and Translational Medicine, University of Tartu, Ravila St. 14b, 50411 Tartu, Estonia; 3 Department of Anatomy, Faculty of Medicine, University of Peradeniya, Peradeniya 20400, Sri Lanka; 4 Institute of Chemistry, University of Tartu, Ravila St. 14a, 50411 Tartu, Estonia; toonika.rinken@ut.ee; 5 Academic Unit of Reproductive and Developmental Medicine, Department of Oncology and Metabolism, Medical School, University of Sheffield, Sheffield S10 2SF, UK

**Keywords:** fluorescence, NTA, lipophilic dyes, extracellular vesicles, zeta potential, detergent

## Abstract

Research on extracellular vesicles (EVs) has intensified over the past decade, including fluorescent membrane labeling of EVs. An optimal fluorescent method requires the size of EVs to be preserved after labeling. Lipophilic fluorescent dyes, such as CellMask™ Green (CMG), have been widely used for this purpose. Here, we investigated conditions affecting the optimum CMG labeling of EVs derived from human choriocarcinoma cells (JAr) and different biological fluids using fluorescence NTA (*fl*-NTA). The effect of CMG labeling on the size, concentration and zeta potential (ZP) on JAr EVs purified with different methods were measured along with biological fluid-derived EVs. With the increase of CMG dye concentration, a significant decrease in the mean size of fluorescent nanoparticles (*fl*-NPs) was observed. The ZP of *fl*-NPs originating from JAr cells with the lowest and highest dye concentrations showed a significant shift towards more and less negative ZP values, respectively. Differences in the concentration of *fl*-NPs were observed for JAr EVs purified using size-exclusion chromatography (SEC) alone and SEC in combination with tangential flow filtration. The proportion of CMG labeling of NPs varied across different biological sources. CMG labeling may be a reliable technique for the detection of EVs using *fl*-NTA.

## 1. Introduction

Extracellular vesicles (EVs) are membrane-bound nanovesicles carrying complex and highly heterogenous cargo [1]. EVs are released from most cell types and can be found in various biological fluids and cell culture conditioned medium [2]. EVs act as mediators of intercellular communications [3] and also as delivery vehicles for exchanging functional biomolecules such as proteins, lipids, and nucleic acids between cells. Hence, EVs are important regulators of different physiological and pathological processes such as signaling and immune system reactions [4]. The current classification of EVs into exosomes and microvesicles is based on EVs biogenesis and site of origin [5]. This further adds an extra layer of complexity to the heterogeneous nature of EVs in terms of size, cells of origin, and surface protein profiles [6,7,8].

Different strategies have been developed for membrane labeling of EVs using fluorescent dye molecules to examine EV uptake into target cells and for tracking of EVs [9,10,11] and to follow their fate once taken up by cells [12]. Various commercially available fluorescent dyes, such as PKH67 [13], DiO [14], DiI [15], DiD [16], DiR [17], CFSE [18], and CellMask™ Green (CMG), have been extensively used for the membrane labeling of EVs. The lipid bilayer of the EV membranes shares a structural resemblance with that of the plasma membrane of cells [19], which facilitates direct or indirect labeling of membranes by lipophilic dyes. Currently, the main application of plasma membrane binding fluorescent probes, with regards to EV staining is to monitor the uptake of EVs by different cells using different forms of fluorescence microscopy [20,21,22]. An example of such an approach is applying fluorescent labeling in staining EVs secreted by cells into the extracellular space that are uptaken and internalized by recipient cells through multiple routes and mechanisms, which involve the fusion with the plasma membrane and an array of endocytic pathways, such as macro/micropinocytosis, phagocytosis, clathrin-dependent endocytosis, and/or (lipid raft) receptor-mediated internalization [3,23].

The most popular quantitative method of EV analysis is performed by nanoparticle tracking analysis (NTA) [24,25,26]. NTA operation is based on the light scattering and the analysis of the Brownian motion of nanoparticles (NPs) to obtain the particle size distribution and particle concentration in a given sample [27,28]. Although NTA working principles and tracking are based on tracking individual particles, it fails to exclude particles which are not of EV origin in its analysis. In other words, the scatter mode of NTA detects the total number of particles in the whole sample irrespective of their origin being an EV or any other kind of NPs. Hence, there is no guarantee that all the NPs detected and reported after NTA analysis in a given sample are of EV origin. This is why labeling EVs with a lipophilic fluorescent (membrane) dye and following them through the use of the fluorescent mode of NTA is regarded potentially as means of distinguishing between NPs of EV’s origin and NPs that are not of EV origin [29]. Although in theory such a simple methodology may be applied to distinguish between NPs of EV origin and non EV origin, no detailed investigations are available to demonstrate that EV staining with a lipophilic fluorescent (membrane) dye may potentially influence different EV characteristics during NTA analysis. This includes different physical properties of EVs such as EV size, concentration, and zeta potential (ZP).

In the current study, we investigated the hypotheses that different conditions such as *fl*-NTA brightness threshold settings, concentration of membrane dye, EV purification method, source of EVs, incubation temperature, and detergent treatment of EVs affect the optimum staining of NPs by a lipophilic fluorescent dye. We used the CellMask™ Green dye which is a commercially available dye belonging to the Cell Mask dye group and being from the lipophilic class of dyes as they stain amphipathic molecules [30]. CMG provides a lipophilic part for efficient membrane loading and a negatively charged hydrophilic section for stably retaining the dye in the plasma membrane. We assessed the effect of CMG labeling on the size, concentration, and ZP values of the labeled NPs using fluorescence-NTA (*fl*-NTA). Furthermore, we compared the differences in physical properties between CMG-labeled NPs in fluorescent and scattering modes.

Our results provide further information about membrane fluorescent labeling for tracking NPs that are presumably from EV origin. However, caution needs to be exercised in attaining this conclusion if the staining protocols used and experimental conditions are not rigorously optimized and standardized. Ultimately, *fl*-NTA has the potential of being used as a simple and practical means of checking the EV sample purity.

## 2. Materials and Methods

### 2.1. Materials

Dulbecco’s PBS (Sigma-Aldrich Co., St. Louis, MO, USA), NP-40 Alternative (Merck KGaA, Darmstadt, Germany), CellMask™ Green plasma membrane stain (Catalog number: C37608, Thermo Fisher Scientific, Altrincham, Greater Manchester, UK)

### 2.2. Cell Culture

The human choriocarcinoma cell line (JAr) from the first trimester trophoblasts was acquired from ATCC^®^ 116 (HTB-144 ™, Teddington, UK). The human choriocarcinoma (JAr) cells were cultured as described previously [31]. Briefly, JAr cells were cultured in a T-75 flask in RPMI 1640 media (Gibco, Paisley, Scotland) supplemented with 1% penicillin/streptomycin (P/S, Gibco 15140122, Bleiswijk, The Netherlands), 1% L-glutamine (Sigma, 59202C, St. Louis, MO, USA), and 10% fetal bovine serum (FBS, Gibco, 10500064) at 37 °C under moist 5% CO_2_-rich conditions. At 80% confluency, the conditioned medium was removed and the cells were washed with 10 mL of unsupplemented RPMI 1640 media to remove traces of FBS. Unsupplemented RPMI medium was replaced with fresh RPMI 1640 medium supplemented with 1% penicillin/streptomycin, 1% L-glutamine, and 10% EV-depleted FBS. Cells were cultured for 24 h at 37 °C and 5% CO_2_. After incubation, the conditioned medium was collected for EV isolation. We have submitted all relevant data of our experiments to the EV-TRACK knowledgebase (EV-TRACK ID: EV190091) [32].

#### EV-Depletion of the FBS

The depletion of EVs in FBS was carried out using a methodology proposed earlier [33]. In brief, FBS was ultrafiltered using Amicon Ultra-15 centrifugal filter devices (100 kDa cutoff, MerkMillipore, Darmstadt, Germany) for 30 min at 5000× *g*. The filtrate was collected and measured for particle concentration using NTA. For this study, the vesicle-depleted conditioned medium was used to isolate and purify EVs by SEC, and the EV preparations met the optimal requirement as per the guidelines prescribed by the International Society for Extracellular Vesicles (ISEV) [34].

### 2.3. EV-Purification from JAr Cell Conditioned Medium

Isolation of EVs by size-exclusion chromatography (SEC) was performed based on prior published methods [35]. The conditioned medium was spun first at 400× *g* for 10 min, while the supernatant from two successive centrifugation steps (4000 and 10,000× *g* for 10 min) to remove cell debris and apoptotic bodies were retained. The collected conditioned medium was concentrated to 500 µL with Amicon Ultra-15 centrifugal filter devices (10 kDa cutoff, MerkMillipore, Darmstadt, Germany) and EVs were isolated from the concentrated media using SEC in a cross-linked 4% agarose matrix of 90 µm beads (Sepharose 4 Fast Flow, GE HealthCare Bio-Sciences AB, Uppsala, Sweden) in a 10 cm gravity column (Econo-Pac^®^ Chromatography columns, Bio-Rad, Hercules, CA, USA) washed and calibrated with PBS. The EV-enriched fractions 6 to 9 (volume 0.5 mL) were collected and concentrated further using Amicon Ultra-15 centrifugal filter devices (10 kDa cutoff), as described previously [31]. Isolated EVs were quantified using NTA (ZetaView, Particle Metrix GmbH, Inning am Ammersee, Germany). Proteomic and electron microscopic characterizations (Figures S1 and S2) of JAr EVs were published in earlier communications [31].

Prior to EV purification by tangential flow filtration (TFF), the conditioned medium underwent sequential centrifugation, as mentioned above. EVs were isolated using a TFF-Easy filtration-based device (HansaBioMed, Tallinn, Estonia). The conditioned medium was concentrated using sterile hollow fibres polysulfone membranes and filtered through 20 nm pores to remove small proteins and free biomolecules. The TFF-Easy filtration device was washed three times with Milli-Q water and the conditioned medium was loaded into pre-connected syringe-1 via the sample injection nozzle of the filtration device. After loading the conditioned medium into pre-connected syringe-1, the conditioned medium was pushed into pre-connected syringe-2 after the closure of a valve on the device. Subsequently, the concentration process of the conditioned medium was begun after opening the permeate nozzles on the device. By pushing syringes 1 and 2 interchangeably upwards and downwards, the conditioned medium was retained and concentrated to 1.5 to 1.9 mL. The collected concentrated conditioned media was transferred to a sterile Eppendorf tube and further concentrated to 500 µL using a 10 kDa cut-off concentrator before subjected to SEC column.

### 2.4. EV-Purification from Bovine Follicular Fluid

The extracellular vesicles from bovine follicular fluid (BFF) were purified based on methods described in the literature [36]. In brief, EVs were isolated from the follicular fluid of ovaries (BFF) obtained from the slaughterhouse (Rakvere, Estonia). Initially, the ovaries were washed three times using PBS supplemented with 1% penicillin-streptomycin and 1% amphotericin B. Then, the BFF was aspired using a vacuum pump (Minitüb GmbH, Tiefenbach, Bavaria, Germany). Initially, samples were centrifuged for 300× *g* for 10 min to remove cells. Subsequently, the supernatant from the previous step was centrifuged at 2000 and 20,000× *g* for 10 and 30 min to remove the cell debris and apoptotic bodies from the follicular fluid. Later, the samples were concentrated up to 500 µL using Amicon^®^ Ultra-15 centrifugal filter units (10 kDa cut-off) (Merck Millipore, Burlington, MA, USA) at 3000× *g* for 1 h at 4 °C. Benchtop SEC columns were used to purify vesicles from the follicular fluid. The vesicle-enriched fractions 5–7 (500 µL each) were collected and concentrated using Amicon^®^ Ultra-15 centrifugal filter units (10 kDa cut-off).

### 2.5. EV-Purification from Seminal Plasma

The extracellular vesicles from seminal plasma were purified based on the method described in the literature [36]. In brief, seminal plasma from three different bulls were pooled and subjected to differential centrifugation. To get rid of cells and cellular debris samples were centrifuged at 500× *g* for 10 min. The supernatant was collected in a clean tube and centrifuged at 2000× *g* for 15 min while maintaining the temperature of the centrifuge 4 °C in all the steps of centrifugation. The supernatant was taken ahead of and centrifuged again for 20,000× *g* for 15 min to remove apoptotic bodies from seminal plasma. Afterwards, the samples were concentrated up to 500 µL using Amicon^®^ Ultra-15 centrifugal filter units (10 kDa cut-off). Benchtop SEC columns were used to purify vesicles from seminal plasma. The vesicle-enriched fractions 5–8 (500 µL each) were collected and concentrated further up to 100 µL using Amicon^®^ Ultra-15 centrifugal filter units (10 kDa cut-off).

### 2.6. CMG Labeling of EVs

#### 2.6.1. JAr EVs Labeling with CMG Dye

JAr EVs purified in SEC and a combination of TFF + SEC were diluted separately in 1 × PBS to a particle concentration of about 1 × 10^10^ particles/mL. Before incubating EVs with CMG dye molecules, 1 µL of 5 mg/mL CMG stock (CellMask™ Green Plasma Membrane Staining, Thermo Fisher Scientific, Waltham, MA, USA) was added to 50 µL of PBS. Then, 1 µL of CMG in 1 × PBS was added to 10 µL diluted EVs and incubated at RT for an hour on a shaker at 350 rpm. Similarly, JAr EVs purified in SEC and labeled with the above-mentioned concentration of CMG dye were incubated at 4, 25, and 37 °C. Additionally, by keeping the concentration of EVs constant, different concentrations of CMG (25 µg/mL, 29.4 µg/mL, and 50 µg/mL) were prepared following the same overall procedure in 1 × PBS. All experimental tubes were kept covered with aluminum foil during incubation. After the incubation, the incubated samples were added to 990 µL of 1 × PBS suspension medium to have a final volume of 1mL with a pH value of 7.2.

#### 2.6.2. CMG Dye Labeling of Lyophilized HCT116 and Biologically Derived EVs

Lyophilized HCT116 CD63 positive EVs (HansaBioMed, Tallinn, Estonia) were reconstituted in MQ water to a protein concentration of 1 ug/uL. Following the manufacturer’s instructions, HCT116 EVs reconstituted in MQ water were diluted in 1 × PBS to a particle concentration of about 1 × 10^10^ particles/mL. In parallel, EVs were purified from both bovine follicular fluid (BFF) and seminal plasma using SEC, as described previously [36], and were diluted in 1 × PBS to the same particle concentration as mentioned above. Before incubating EVs with CMG dye molecules, 1 µL of 5 mg/mL CMG stock (CellMask™ Green Plasma Membrane Staining, Thermo Fisher Scientific) was added to 50 µL of PBS. Then, 1 µL of CMG in 1 × PBS was added to 10 µL diluted EVs and incubated at RT for an hour on a shaker at 350 rpm. All the experimental tubes were kept covered with aluminum foil during incubation. After the incubation, the incubated samples were added to 990 µL of 1 × PBS suspension medium to have a final volume of 1mL with a pH value of 7.2.

### 2.7. NP-40 Detergent Treatment of Neat (Unlabeled) and CMG-Labeled EVs

For detergent treatment of nanoparticles, EVs purified in SEC were diluted in 1 × PBS to a particle concentration of about 1 × 10^10^ particles/mL. Before incubating EVs with and without CMG dye molecules, 1 µL of 5 mg/mL CMG stock (CellMask™ Green Plasma Membrane Staining, Thermo Fisher Scientific) was added to 50 µL of 1 × PBS. Then, 1 µL of CMG in 1 × PBS was added to 10 µL diluted EVs. Controls for detergent treatments included detergent only and EVs without detergent (NP-40). For NP-40 detergent treatment controls, EVs without or with CMG dye were treated with a final concentration of 0.5% NP-40 detergent in a 10 µL reaction volume. (Note: We tried different concentrations such as 0.01, 0.1, 0.5, 1 and 2% of NP-40; amongst them, we found that EVs’ lipid membrane was disrupted and saturation was reached at 0.5% concentration of NP-40. Samples were incubated for an hour on a Biosan TS-100 Thermo- shaker set at RT (25 °C) and speed of 350 rpm. Note that all experimental tubes were covered with aluminum foil during incubation. After the incubation, the incubated samples were added to 990 µL of 1 × PBS suspension medium, to have a final volume of 1mL with a pH value of 7.2, resulting in a sample with final concentrations of about 1 × 10^8^ particles/mL EVs and 100 ng/mL of CMG. The size, concentration, and ZP of EVs were measured both in scatter and fluorescent modes as described below in the manuscript.

### 2.8. Nanoparticle Tracking Analaysis of EVs

Nanoparticle tracking analysis (NTA) was performed using a ZetaView PMX 120 V4.1 instrument (Particle Metrix GmbH, Ammersee, Bavaria, Germany). The instrument was calibrated using a known concentration of 100 nm polystyrene (PS) and fluorescent Yellow-Green (YG) nanoparticles (Applied Microspheres B.V., Leusden, Utrecht, The Netherlands). The standards were suspended in particle-free water, whereas the EV samples were diluted with 1 × PBS for analyses. Particle number and size distribution were counted at 11 frames per cycle under a sensitivity of 72 and a shutter value of 100.

### 2.9. Zeta Potential Measurements

The zeta potential (ZP) of EVs was measured three times at 25 °C under the following scatter and fluorescent settings: Sensitivity was set at 72, shutter value at 100, and frame rate at 30 frames per second. For the size and ZP measurement of fluorescently labeled EVs, the sensitivity was set at 90.

### 2.10. Statistical Analysis

Statistical analyses were done using Graphpad prism v8.4.2. The comparison between the concentration and ZP of fluorescently labeled and total particles were assessed using the two-tailed Student *t*-test. The effects of different conditions were carried out using one-way or two-way ANOVAs. Tukey’s multiple comparison tests were applied for specific intergroup comparisons. Data are shown as mean ± SD (*n* = 3). Differences were taken as statistically significant at *p* ≤ 0.05 and were marked with an asterisk (*) symbol.

### 2.11. Experimental Design

The experiments were designed to test the validity of the following hypotheses:

- The fl-NTA brightness threshold affects the detection of fluorescent NPs in a given sample.

Experiments were designed to determine the effect of minimum brightness threshold settings on size, concentration, and ZP values of fluorescent NPs (*fl*-NPs) compared to total NPs (*t*-NPs) present in a given sample. The effect of *fl*-NTA brightness detection threshold settings on the measurement of size, concentration, and ZP of *fl*-NPs originating from the JAr and HCT116 cells were measured at two different minimum threshold brightness values, at 25 and 30, using ZetaView^®^ software. All other post-acquisition settings remained unchanged. Experiments were performed on three different days and each day three technical measurements were obtained from each sample resulting in a total number of 9 size and ZP measurements values (*n* = 9). ZetaView^®^ NTA was used to measure the difference in physical properties between CMG-labeled and scattered NPs originated from the above-mentioned cells. Hereafter, CMG-labeled and scattered NPs are termed fluorescent NPs (*fl*-NPs) and total NPs (*t*-NPs) respectively.

II. The CMG dye concentration affects the size distribution, concentration, and ZP values of *fl*-NPs.

Experiments were performed to evaluate the effect of CMG dye concentration on the size, concentration, and ZP values of *fl*-NPs compared to *t*-NPs present in a given sample. The effect of CMG concentration on the physical properties of *fl*-NPs originating from the JAr cells was studied at a varied concentration of CMG while keeping the concentration of EVs constant. Henceforth, a minimum threshold brightness value of 25 was maintained all the time for the measurements of *fl*-NPs in the fluorescent mode of NTA. All the respective pre and post-acquisition settings for scattering and fluorescent modes of NTA remained unchanged. Experiments were performed on three different days and each day three technical measurements were obtained from each sample resulting in a total number of 9 size and ZP measurements values (*n* = 9).

III. Incubation temperature affects the proportion of *fl*-NPs in a given sample.

Experiments were performed to evaluate the effect of incubation temperature on the size, concentration, and ZP values of *fl*-NPs in comparison to *t*-NPs present in a given sample. The effect of incubation temperature on *fl*-NPs was measured for JAr EVs that were incubated at 4, 25, and 37 °C. After the incubation, EV samples were adapted to room temperature before NTA measurements. Experiments were performed in triplicate and the size, concentration, and ZP of *fl*-NPs and *t*-NPs of the incubated samples were measured as mentioned elsewhere.

IV. The EV purification method affects the proportion of CMG positive *fl*-NPs present in a given sample.

Experiments were done to test the effect of EV purification method on the size, concentration, and ZP values of *fl*-NPs compared to *t*-NPs present in a given sample. The effect of EV purification on the physical characteristics of *fl*-NPs was measured using ZetaView^®^ NTA for JAr EVs purified in SEC alone and a combination of TFF. Experiments were performed in triplicate and the size, concentration, and ZP of *fl*-NPs and *t*-NPs purified in SEC alone and a combination of TFF were measured as described elsewhere.

V. Detergent treatment of EV’s membrane affects the proportion of *fl*-NPs in a given sample.

Experiments were performed to confirm the disruption of EVs’ lipid bilayer membrane using a nonionic detergent NP-40 in the absence and presence of CMG dye molecules within a given sample. Detergent treatment of NPs was performed for JAr EVs both in the absence and presence of CMG dye molecules. Experiments were performed in triplicate and the size and concentration of EVs before and after NP-40 detergent treatment as well as NP-40 treated *fl*-NPs and *t*-NPs were measured both in scatter and fluorescent modes, as mentioned elsewhere.

VI. The source of material for EV purification affects *fl*-NPs proportions to the total number of NPs present in a given sample.

Experiments were performed to determine the effect of the EV source on the size, concentration, and ZP values of *fl*-NPs compared to *t*-NPs present in a given sample. EVs were purified from bovine follicular fluid (BFF) and seminal plasma using SEC. Experiments were performed in triplicate and the size, concentration, and ZP of biologically-derived NPs were measured both in scatter and fluorescent modes of ZetaView^®^ NTA, as described elsewhere.

## 3. Results

### 3.1. Effects of Minimum Brightness Threshold Settings on the Detection of Fluorescent Nanoparticles

To determine the effect of minimum brightness threshold settings on size, concentration, and ZP values of CMG-labeled NPs, *fl*-NPs were measured at two different minimum brightness threshold values, of 25 and 30, using ZetaView^®^ NTA software. The fluorescent threshold settings affected the size distribution, concentration, and ZP of *fl*-NPs. Optimized settings for fluorescently labeled NPs were reached after measuring *fl*-NPs at two different minimum brightness threshold values of 25 and 30.

**Figure 1:**
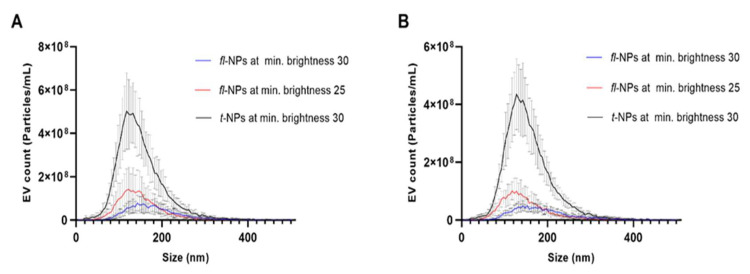
Size profiles of CMG labeled fluorescent and total NPs originated from JAr and HCT116 cells measured at 30 and 25 minimum brightness threshold values using Zetaview^®^-NTA. Size profile distribution for CMG-labeled *fl*-NPs and *t*-NPs of JAr (**A**) and HCT 116 (**B**) EVs diluted in 1 × PBS at scattering and fluorescent NTA measurements. (Mean ± SD, *n* = 9).

The presence of EVs with heterogeneity in sizes was observed both in CMG-labeled EVs measured in scattered (i.e., total) and fluorescent modes of NTA (Figure 1). Fluorescent NPs originating from JAr cells measured at 25 minimum brightness showed a high particle distribution in the range of ~50 to 180 than the size distribution assessed at 30 minimum brightness (Figure 1A). A similar result was observed for fluorescent NPs originating from HCT 116 cells (Figure 1B). Overall, in Figure 1, there is a clear trend of increase in the *fl*-NPs for JAr and HCT 116 EVs measured at a minimum brightness value of 25.

**Figure 2:**
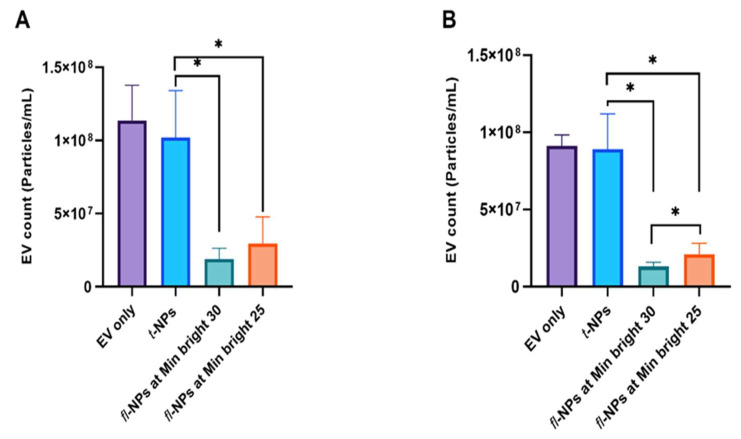
Concentration of total and fluorescent NPs originated from JAr and HCT 116 cells. The concentrations for EVs only control (light purple boxes), CMG-labeled fluorescent (cyanish-green and orange boxes), and total NPs (light blue boxes) of JAr (**A**) and HCT 116 EVs (**B**), measured in fluorescent and scatter modes of ZetaView^®^ NTA at two different minimum brightness threshold levels. The data points for *fl-*NPs measured at two different minimum brightness threshold levels were significantly different (*p* ≤ 0.05) than *t*-NPs measured at the highest brightness threshold, that is, 30 and thus were marked with an asterisk (*) symbol (mean ± SD, *n* = 9).

The effect of the minimum brightness threshold level used during fluorescence measurement of particles has a detrimental impact on the concentration of *fl*-NPs and the final output of fluorescence signals (Figure 2). The concentration of *fl*-NPs originating from JAr cells measured at low brightness (~30%) has provided more fluorescently labeled particles than *fl*-NPs measured at a high brightness threshold value (~19%) (Figure 2A). In other words, there was a significant difference between total and fluorescent NPs measured at a minimum brightness threshold of 25 (*p* ≤ 0.05) and 30 (*p* ≤ 0.05). Regarding HCT 116 EVs, there was also a significant difference between the two different minimum brightness threshold levels used for the detection of *fl*-NPs (Figure 2B). The results showed that, at a low brightness threshold level, *fl*-NPs were detected more (~24%) than the ones measured at a high brightness threshold level (~15%). The difference between the respective CMG-labeled NPs originating from HCT 116 cells measured in scattered and fluorescent modes of NTA was significant at minimum brightness threshold values of 25 (*p* ≤ 0.05) and 30 (*p* ≤ 0.05).

**Figure 3:**
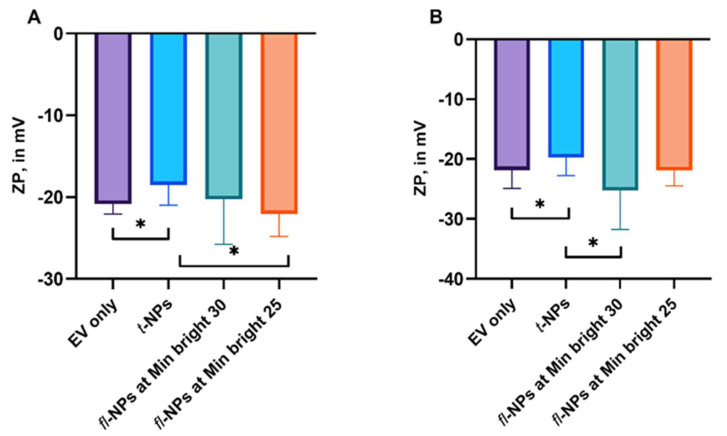
ZP measurement of total and fluorescent NPs for JAr and HCT 116 EVs labeled with CMG dye. The ZP for EVs only control (light purple boxes), CMG-labeled fluorescent (cyanish-green and orange boxes) and total NPs (light blue boxes) of JAr (**A**) and HCT 116 EVs (**B**) measured in the fluorescent and scattered mode of NTA at two different minimum brightness threshold levels. The ZP values of *fl-*NPs originating from JAr and HCT116 cells measured at minimum brightness threshold of 25 and 30 were significantly different (*p* ≤ 0.05) than the respective *t*-NPs originating from JAr and HCT116 cells, thus were marked with an asterisk (*) symbol. EVs were diluted in 1x PBS, and the ZP measurement was done at a neutral pH of 7.2 (mean ± SD, *n* = 9).

The impact of the minimum brightness threshold level used during the fluorescence measurement of ZP of fluorescent and total NPs originating from JAr and HCT 116 cells were also assessed by Zetaview^®^-NTA (Figure 3). The ZP of *fl-*NPs originating from JAr cells measured at a low brightness threshold level (*p* ≤ 0.05) showed a significant shift towards a more negative value, than *fl-*NPs measured at a high brightness threshold level (*p* = 0.381). These comparisons were made to their respective CMG-labeled *t*-NPs of JAr EVs measured in scatter mode.

The ZP of *fl-*NPs originating from HCT 116 cells measured at a high brightness threshold value (*p* ≤ 0.05) showed a significant shift towards a more negative value, than *fl-*NPs measured at a low brightness threshold level (*p* = 0.072). The above-mentioned comparisons were made for CMG-labeled *t*-NPs of HCT EVs measured in scatter mode. Similar to JAr EVs, the addition of CMG dye on HCT 116 EVs induced a significant shift towards less negative ZP values for CMG-labeled *t*-NPs measured in scatter mode (*p* ≤ 0.05).

### 3.2. Effect of CMG Concentration on the Size Distribution, Concentration and ZP Values of fl-NPs of EVs

**Figure 4:**
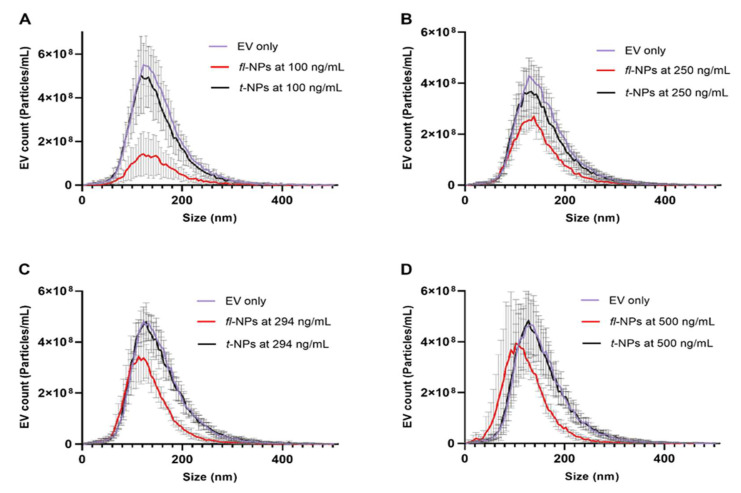
Effect of CMG concentration on the size profile distribution of fluorescent and total NPs of EVs measured using Zetaview^®^- NTA. (**A**–**D**) Size profile distribution for *fl-*NPs originating from JAr cells measured at a minimum brightness threshold value of 25 with different CMG concentrations of 100, 250, 294 and 500 ng/mL respectively. EVs only as a control, *fl* and *t*-NPs of EVs diluted in 1× PBS and measured in fluorescent and scatter modes of NTA (mean ± SD, *n* = 9).

To evaluate the effect of CMG concentration on the size distribution, particles mean size, concentration, and ZP values of *fl-*NPs originating from JAr cells, the particle’s size distribution was measured using Zetaview^®^ NTA. By keeping the concentration of EVs constant (Video S1), the concentration of CMG dye was varied accordingly. As it was shown in the previous section of the result, EV heterogeneity in sizes was also observed for EVs. The size distribution for EV only control (i.e., Scatter), and EVs labeled with CMG (Videos S2 and S3), which was measured in fluorescent (i.e., *fl-*NPs) and scatter (i.e., total t-NPs) modes of NTA, have shown size range values of ~30–400 nm (Figure 4). Fluorescent NPs originating from JAr cells showed a high particle distribution in the range of ~40/60 to 260/280 nm as the concentration of CMG dye increased. Moreover, the size distribution shift towards smaller particles was detected for *fl-*NPs when the CMG concentration increased. Surprisingly, CMG nanoparticles were not detected in the CMG only control, both in the scatter and fluorescent modes of NTA.

**Figure 5:**
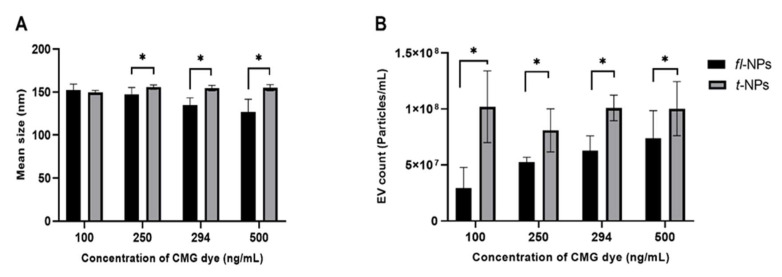
The mean size distribution and concentration for JAr EVs labeled with CellMask™ Green (CMG). All EV types were labeled with different concentrations of CMG (100, 250, 294 and 500 ng/mL respectively). Particle means size (**A**) and concentration (**B**) for respective *fl* and *t*-NPs originating from JAr cells measured in fluorescent and scatter modes. The mean particle size and concentration of *fl*-NPs were significantly different (*p* ≤ 0.05) than *t*-NPs at all concentrations of CMG, except the mean particle size comparison between *fl*-NPs and *t*-NPs at the lowest concentration of CMG. Thus, were marked with an asterisk (*) symbol (mean ± SD, *n* = 9).

Quantitative determination on the means size of total and *fl-*NPs of JAr EVs (Figure 5A) showed a significant shift in the means towards smaller particles size with successive increases in CMG dye concentration (*p* ≤ 0.05), except for the lowest dye concentration (*p* = 0.354). Similarly, JAr EVs labeled with varying concentrations of CMG dye showed a difference in the concentration of *fl-*NPs. The concentration of *fl-*NPs of JAr EVs at a higher concentration of dye provided more fluorescently labeled particles than EVs labeled with the lowest concentration of dye (Figure 5B). T-test found a significant difference in the concentration of total and *fl-*NPs originating from JAr cells labeled with different concentrations of CMG dye (*p* ≤ 0.05).

**Figure 6:**
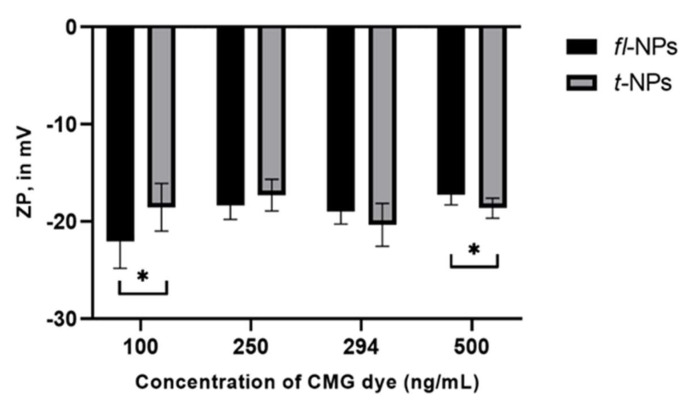
ZP measurement of fluorescent and total NPs originating from JAr cells labeled with different concentrations of CMG dye (100, 250, 294 and 500 ng/mL respectively). The ZP values for *fl* and *t*-NPs at the lowest and highest concentrations of CMG were significantly different (*p* ≤ 0.05) than the corresponding *t*-NPs at 100 and 500 ng/mL of CMG dye. Thus, were marked with an asterisk (*) symbol. JAr EVs measured in fluorescent and scatter modes of NTA. EVs were diluted in 1× PBS, and the ZP measurement was done at a neutral pH of 7.2 (mean ± SD, *n* = 9).

The effect of CMG concentration on the ZP of *t*- and *fl*-NPs originating from JAr cells was also assessed by ZetaView^®^-NTA (Figure 6). The ZP of *fl*-NPs of JAr EVs with the lowest and highest dye concentrations showed a significant shift towards more (*p* ≤ 0.05) and less (*p* ≤ 0.05) negative ZP values, respectively.

### 3.3. Effect of Incubation Temperature on the Physical Characteristics of fl-NPs of EVs

**Figure 7:**
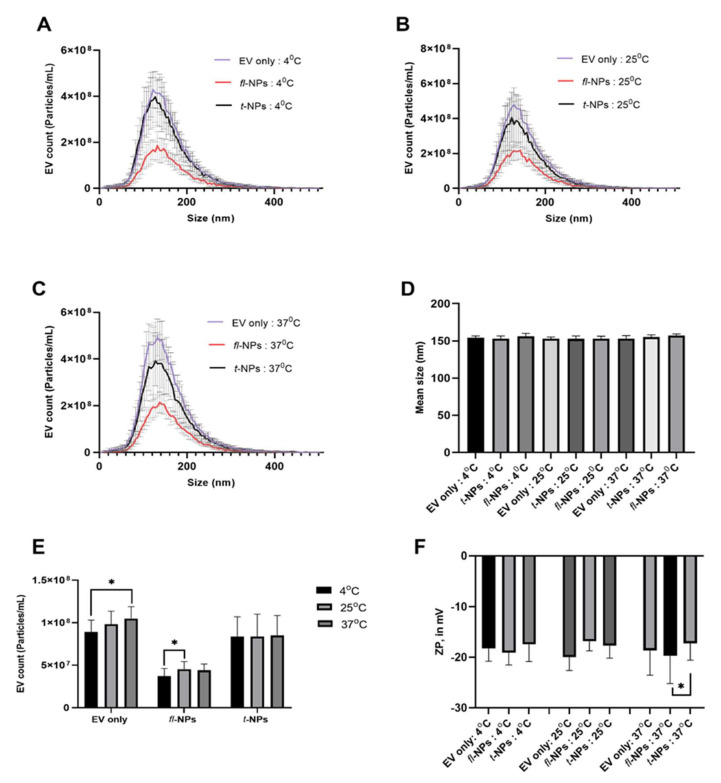
The size distribution, concentration, and zeta potential of fluorescent and total NPs originating from JAr cells incubated at three different temperatures. Size distribution (**A**–**C**), means particle size (**D**), particle concentration (**E**), and zeta potential (**F**) of EVs incubated at 4, 25, and 37 °C. EV only controls, *fl*-, and *t-*NPs of JAr EVs were measured in fluorescent and scatter modes of NTA. EVs were labeled with 100 ng/mL of CMG and *fl*-NPs were measured at a minimum brightness threshold value of 25. The concentration of *fl*-NPs of JAr EVs incubated at 4 °C was significantly different (*p* ≤ 0.05) than the *fl*-NPs of JAr EVs incubated at 25 °C. Thus, were marked with an asterisk (*) symbol. EVs were diluted in 1× PBS, and the ZP measurement was done at a neutral pH of 7.2 (mean ± SD, *n* = 9).

To study the effect of incubation temperature on the size, concentration, and ZP values of *fl*-NPs originating from JAr cells, JAr EVs were kept at three different incubation temperatures of 4, 25, and 37 °C. EVs heterogeneity in size distributions was also observed for EVs incubated at these temperatures (Figure 7A–D). JAr EVs incubated at the lowest temperature showed a significant decrement in the concentration of *fl-*NPs in comparison to *fl*-NPs incubated at 25 °C (*p* ≤ 0.05). Otherwise, the effect of incubation temperature was not observed between the concentration of *fl*-NPs incubated at 25 and 37 °C (Figure 7E). JAr EVs incubated at the two highest temperatures provided slightly higher *fl-*NPs than EVs incubated at the lowest temperature. The ZP of EV only, *fl*-, and *t*-NPs were also measured for EVs incubated at these incubation temperatures. The results, as shown in Figure 7F, indicate that the ZP of *fl*-NPs incubated at 37 °C exhibited a significant shift towards a more negative value than *t*-NPs incubated at the same temperature (*p* ≤ 0.05). Whereas a non-significant difference in ZP values of *fl* and *t*-NPs was observed for EVs incubated at 4 and 25 °C. Moreover, the effect of incubation temperature did not tend to cause a significant difference in ZP values for EV only samples incubated at different temperatures.

### 3.4. Effect of EV Purification on the Physical Characteristics of fl-NPs of EVs

**Figure 8:**
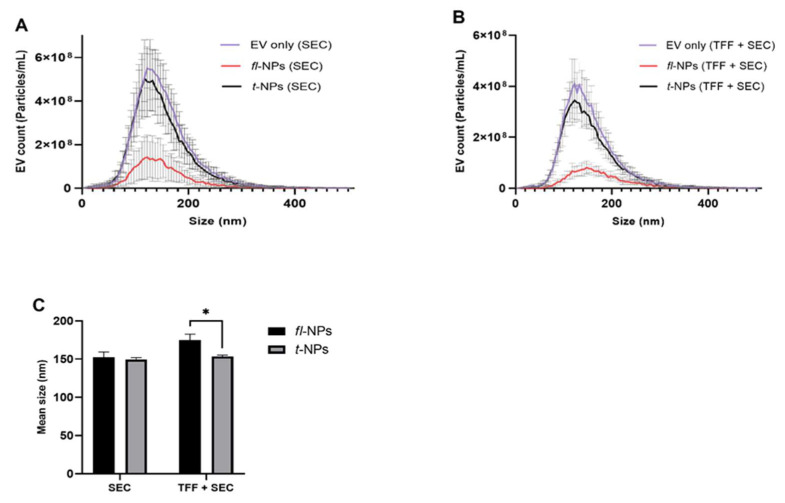
Size profile distribution for fluorescent and total NPs originating from JAr cells measured using Zetaview^®^- NTA. Size profile distribution (**A**,**B**) and particle mean size (**C**) for JAr EVs purified in SEC alone and combination of TFF. EVs were labeled with 100 ng/mL of CMG and *fl-*NPs were measured at a minimum brightness threshold value of 25. The mean particle size of *fl-*NPs of JAr EVs purified with TFF and SEC was significantly different (*p* ≤ 0.05) than the corresponding t-NPs of JAr EVs purified with TFF and SEC. Thus, were marked with an asterisk (*) symbol. EVs only as a control, *fl-*, and t-NPs of EVs were diluted in 1x PBS before being measured in fluorescent and scatter modes of NTA (mean ± SD, *n* = 9).

To evaluate the effect of the EV purification method on the physical characteristics of *fl*-NPs of EVs, JAr EVs were purified with SEC alone and in a combination of TFF. EV heterogeneity in sizes was observed for EVs purified in SEC and a combination of TFF. The size distribution for *fl*-NPs of JAr EVs purified in TFF and SEC showed a high particle distribution in the range of ~60 to 280 nm. Interestingly, the size shift towards larger particles was detected more for *fl*-NPs of JAr EVs purified in TFF and SEC than SEC alone (Figure 8B). Further statistical analysis on the mean particle size of fluorescent and t-NPs of JAr EVs purified in TFF plus SEC showed a significant shift towards larger particles (*p* ≤ 0.05). In contrast, the mean particle size of JAr EVs purified in SEC alone was not different in *fl*-NPs and *t*-NPs (*p* = 0.354).

**Figure 9:**
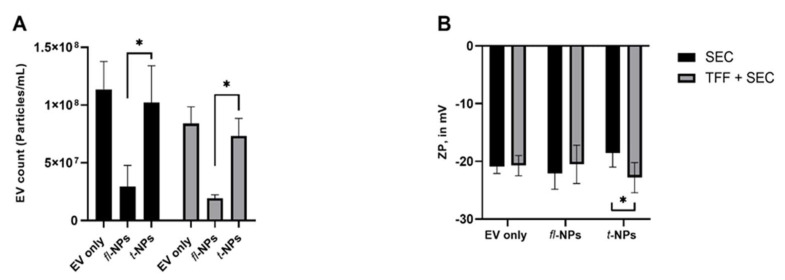
Concentration and zeta potential of fluorescent and total NPs for JAr EVs purified in SEC alone and combination of TFF. Particle concentration (**A**) and zeta potential (**B**) for respective EV only controls, *fl-*, and t-NPs of JAr EVs measured in fluorescent and scatter modes of NTA. All EV types were labeled with 100 ng/mL of CMG and *fl-*NPs were measured at a minimum brightness threshold value of 25. The concentration of *fl-*NPs of JAr EVs purified in SEC and in combination with TFF was significantly different (*p* ≤ 0.05) than the corresponding t-NPs of JAr EVs purified with SEC alone and in combination with TFF. Thus, were marked with an asterisk (*) symbol. EVs were diluted in 1× PBS, and the ZP measurement was done at a neutral pH of 7.2 (mean ± SD, *n* = 9).

JAr EVs purified in SEC alone and the combination with TFF showed a difference in the concentration of *fl-*NPs. JAr EVs purified in SEC provided more *fl-*NPs than EVs purified with TFF plus SEC. The labeling efficiency for CMG-labeled EVs purified in SEC and in combination with TFF was ~30% and ~26%, respectively. The ZP of *fl-* and t-NPs was measured for EVs purified both in SEC only and in the combination of TFF. The results, as shown in Figure 9B, indicate that the ZP of *fl-*NPs of JAr EVs purified in SEC exhibited a significant shift towards more negative values than respective t-NPs. Whereas a non-significant difference in ZP values for *fl-* and t-NPs was observed for EVs purified in the combination of TFF and SEC (*p* = 0.183). Besides, the ZP of t-NPs of JAr EVs purified in the combination of TFF and SEC showed a more negative value than the ZP of t-NPs of JAr EVs purified in SEC alone (*p* ≤ 0.05). Otherwise, the effect of the EV purification methods did not cause any significant differences in ZP values for EV only (control) and *fl-*NPs of JAr EVs purified in SEC alone and a combination of TFF.

### 3.5. Effect of Detergent on EV Lipid Bilayer on the Context of Fluorescent EV

**Figure 10:**
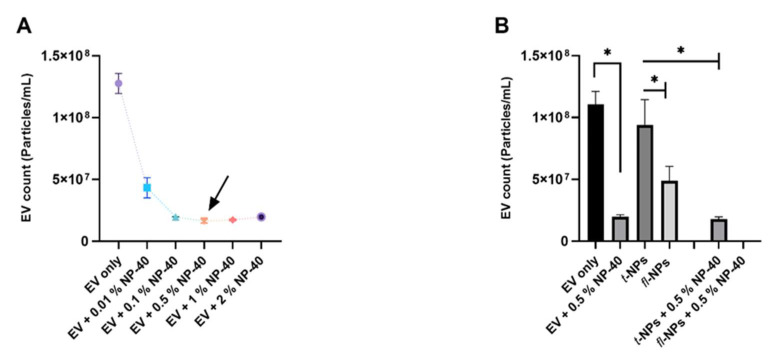
Concentration of fluorescent and total particles originating from JAr cells after NP-40 detergent treatment. EVs were labeled with 100 ng/mL of CMG and *fl-*NPs were measured at a minimum brightness threshold value of 25. (**A**) EVs were treated with different concentrations (0.01%, 0.1%, 0.5%, 1%, and 2%) of NP-40. EVs membrane was disrupted and saturation was reached at 0.5% concentration of NP-40. (**B**) EVs with or without CMG dye were treated with 0.5% of NP-40 and their respective particle concentration. The concentration of t-NPs of JAr EVs before 0.5% of NP-40 treatment was significantly different (*p* ≤ 0.05) than the t-NPs of JAr EVs after NP-40 treatment. Thus, were marked with an asterisk (*) symbol. EV only controls, *fl-*, and t-NPs JAr EVs were diluted in 1x PBS and particle concentration was measured in fluorescent and scatter modes of NTA. (Mean ± SD, *n* = 9).

To confirm that the particles detected were surrounded by a lipid bilayer (membrane), EVs were treated with a nonionic 0.5% NP-40 detergent to disrupt the membrane. NP-40 detergent treatment of EVs without the presence of CMG dye almost eliminated detected particles (*p* ≤ 0.05). Following the addition of NP-40 detergent into CMG-labeled EVs, a significant decrease in the concentration of t-NPs was observed (*p* ≤ 0.05). This result also showed complete membrane disruption of *fl-*NPs upon detergent treatment (Figure 10B).

### 3.6. Effect of Source of EVs on the Fluorescent EV Proportions

**Figure 11:**
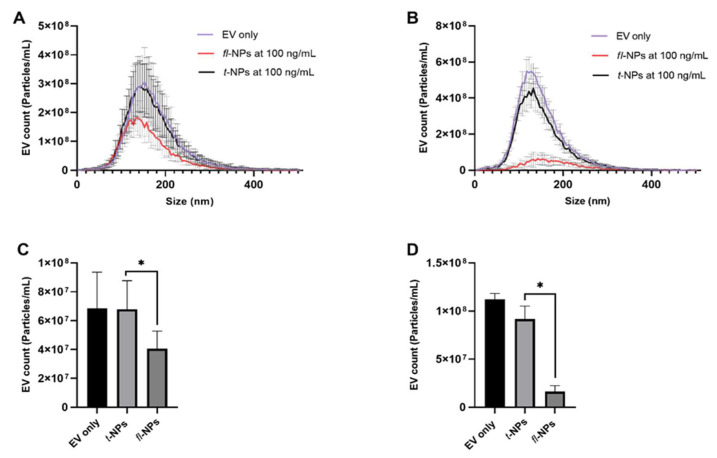
The size distribution and concentration of fluorescent and total NPs for BFF and seminal plasma-derived EVs purified with SEC columns. EVs were labeled with 100 ng/mL of CMG and *fl-*NPs were measured at a minimum brightness threshold value of 25. Size distribution (**A**,**B**), and particle concentration (**C**,**D**) of BFF and seminal plasma-derived EVs incubated at 25 °C respectively. The concentration of *fl-*NPs of BFF and seminal plasma-derived EVs were significantly different (*p* ≤ 0.05) than the corresponding t-NPs of BFF and seminal plasma-derived EVs. Thus, were marked with an asterisk (*) symbol. EV only controls, *fl-*, and t-NPs of BFF and seminal plasma-derived EVs were measured at fluorescent and scatter mode of NTA. EVs were diluted in 1× PBS, and the particle concentration measurement was done at a neutral pH of 7.2 (mean ± SD, *n* = 9).

To determine the effect of the source of EVs on the physical characteristics of *fl-*NPs, EVs were purified from bovine follicular fluid (BFF) and seminal plasma using SEC columns. EVs heterogeneity in size was also observed for these SEC- purified biologically-derived vesicles. The size distribution for *fl-*NPs of BFF EVs showed a high particle distribution in the range of ~33 to 380 nm. Whereas *fl-*NPs of seminal plasma-derived EVs fall under the size distribution range of ~50 to 380 nm. In Figure 11A there is a clear trend of size shift towards smaller particles for *fl-*NPs of BFF EVs. The difference in concentration of *fl-*NPs between BFF and seminal plasma-derived EVs were highlighted in Figure 11C,D. The labeling efficiency of EVs with CMG was higher in BFF EVs (~60%) than EVs purified from seminal plasma (~18%).

**Figure 12:**
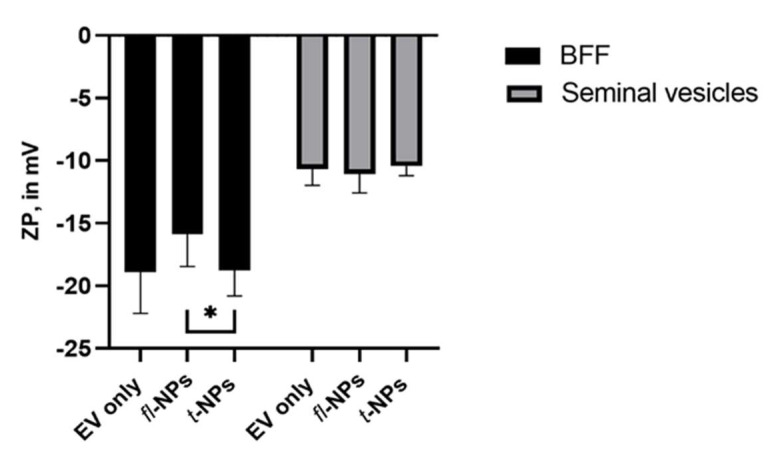
Zeta potential of fluorescent and total NPs for BFF and seminal plasma-derived EVs purified in SEC. All EV types were labeled with 100 ng/mL of CMG and *fl-*NPs were measured at a minimum brightness threshold value of 25. The ZP value for *fl-*NPs of BFF EVs was significantly different (*p* ≤ 0.05) than the corresponding t-NPs of BFF. Thus, were marked with an asterisk (*) symbol. The zeta potential for respective EV only controls, *fl-*, and t-NPs of BFF and seminal plasma-derived EVs measured in fluorescent and scatter modes of NTA. EVs were diluted in 1× PBS, and the ZP measurement was done at a neutral pH of 7.2 (mean ± SD, *n* = 9).

The ZP of fl and t-NPs was measured for BFF and seminal plasma-derived EVs (Figure 12). The ZP of *fl-*NPs of BFF EVs purified using SEC was significantly shifted towards less negative values than respective t-NPs (*p* ≤ 0.05). Whereas a non-significant difference in ZP values of fl and t-NPs was observed for seminal plasma-derived EVs (*p* = 0.353).

## 4. Discussion

The results showed that size, concentration, and ZP, measuring values of fluorescent NPs originated from JAr cells, were dependent on the brightness threshold values set for the camera of the *fl*-NTA instrument. This was also true for fluorescent NPs originated from HCT116 cells. In *fl*-NTA, it is very important to identify the optimum level of the camera’s sensitivity threshold, at which the smallest NPs in a heterogeneous sample would be adequately illuminated and visible to the camera. Whereas an increase in the minimum brightness detection setting of the camera will lead to the detection of only large and bright NPs, which will hide the smaller NPs. On the other hand, lowering the brightness setting of the camera will increase the detection of the background noise and registering noise as *fl*-NPs. Hence it is advisable to have an optimized range of the brightness threshold level for proper analysis and detection of fluorescently labeled particles in a given EV sample [37]. Availability of a calibration standard with the pre-measured size of nanoparticles with a certain size and fluorescence intensity will help to set a optimized brightness level for the given instrument before starting to use the instrument for successive measurements and analysis of different biological samples.

A successive increase in CMG dye concentration led to the decrease of the detected *fl*-NP mean size (Figure 4). At the highest concentration of CMG dye, *fl*-NP sizes in the range of approximately 15 to 40 nm were detected. Such NPs were not detected when the scatter mode was used. These results reflect those of Carnell-Morris et al., who used CMO (CellMask™ Orange) to label plasma EVs. Their *fl*-NTA data showed also the detection of *fl*-NPs at a size range of approximately 15 to 30 nm that were not detected when scatter mode detection was employed [29]. Although in another investigation, Shimomura et al. studied exosomes stained with Mem and PKH dyes where the size of the Mem-labeled exosomes remained constant [38]. This observation was similar to our observation when the lowest concentration of CMG was used for EV labeling and detection of *fl*-NPs. Contrary to our findings, Dehghani et.al reported that increasing PKH dye concentrations led to an increase in the size of *fl*-NPs detected by *fl*-NTA that can be the results of artefacts produced by aggregated PKH molecules [39]. Such differences in the number of NPs measured by scatter or fluorescent modes might have occurred due to several factors, such as the number of non-EV particles in a given sample, the difference in structural design of the dye used for EV labeling, for example, its hydrophilicity and anchoring ability, type and nature of EVs used, along with the NP concentration, incubation temperature, etc. [40]. However, we have no explanation for the size differences between scatter and *fl*-NTA measurements at the highest CMG concentrations. The existence of such differences prioritize optimization of dye concentrations to assure a similar size profile between fluorescent and scatter modes of NTA.

It is known that lipophilic dyes are more fluorescent in a lipidic environment than in an aqueous environment and carry a negative surface charge [41]. For instance, we observed that the ZP of *fl*-NPs originating from JAr cells showed a significant shift towards more and less negative ZP values with the lowest and highest dye concentrations, respectively. This change in ZP values of *fl*-NPs was mainly caused by the presence of molecular interactions between dye molecules and EVs’ membrane. In other words, the surface charge of a membrane is crucial to the membrane permeability (performance), as membrane surface charge influences the electrostatic repulsive forces between charged dye molecules and EV’s membrane surface. Furthermore, due to the amphoteric nature of the CMG dye used in this study, the surface charge (ZP) of the EV-dye complex might have been slightly altered upon masking of EV membrane binding sites at the highest dye concentration. Thus, such differences in net surface charge were observed for the ZP of *fl*-NPs at the above-mentioned concentrations of dye. There are some similarities between the ZP values of *fl*-NPs originated from JAr cells and those described by Shimomura et al., who also reported similar ZP values of Mem dye-stained exosomes [38].

Fl-NTA can potentially be used as a simple methodology to determine the proportion of EVs in a sample of NPs. An EV purification index can be calculated as the ratio of the concentration of NPs measured at the fluorescence mode to that of NPs measured at the scatter mode. This can be a simple index to compare the efficiency of different methods of EV purification techniques like the example used in the current investigation, that is, a comparison between the EV purification efficiency of tissue culture media using SEC alone or SEC in combination with TFF showed that, after SEC, 29% of NPs were fluorescent, in comparison to 24% after EV purification using a combination of SEC and TFF. Although one was expecting a higher purity efficiency after TFF + SEC in comparison to SEC alone, the main difference was in the size profile of EVs (*fl*-NPs), which had a bigger average size in TFF + SEC compared to SEC alone. Such information is completely obscured if one only pays attention to scatter mode NTA and would not have access to *fl*-NTA data. Such a simple index (i.e., EV purification based on the ratio of the concentration of NPs measured at the fluorescence mode to that of NPs measured at the scatter mode) can be a practical mean of establishing the purity of the sample as well as the efficiency of different methodologies for EV purification.

Temperature-related differences in the CMG-labeling ratios were slightly higher for EVs incubated at the two highest incubation temperatures (25 and 37 °C) compared to EVs incubated at 4 °C (Figure 7E). Most probably, an increase in the incubation temperature led to a higher fluidity of the EV’s lipid bilayer and favored the intercalation of CMG dye molecules into lipid bilayers. Thus, CMG tends to label EVs at a slightly faster pace at higher incubation temperatures. This indicates that the labeling ratios for *fl*-NPs of EVs were less affected by different incubation temperatures. Although, in another investigation, using high-resolution flow cytometry, Tertel et al. studied the impact of incubation temperature on labeling of enhanced GFP EVs with different fluorescence-conjugated antibodies [42]. This observation and our study showed generally higher labeling efficiency of EVs at the highest incubation temperature, though the detection methods employed were different. Thus, evaluation of the effect of temperature on labeled EVs while using different EV detection methods is recommended, in order to obtain consistent and reproducible results.

Moreover, the membrane integrity of the JAr EVs was confirmed using non-ionic detergent, such as NP-40. Detergent treatment of neat (unlabeled) and CMG-labeled EVs led to a significant loss in NPs concentration originating from JAr cells. Complete removal of NP-40-treated *fl*-NPs from detected particles demonstrated that the NPs under study were enclosed with a lipid bilayer membranous structure. Comparably, different studies reported the effect of detergents on the stability of EVs [43,44]. It is well-known that non-ionic detergents have high propensity to break lipid–lipid and lipid–protein interactions compared to protein–protein interactions [45]. Such detergents can exert an imbalance of forces at the hydrophobic-hydrophilic surfaces on the NPs, thus affecting the EVs membrane. These types of conditions clearly demonstrate that the particles under this study were bound with lipid bilayer membranous structures.

Biological EV sources tended to behave slightly differently in their size distribution but varied highly in terms of their CMG labeling ratios in comparison to cell culture-derived EVs. This labeling ratio variation might be caused by the difference in the composition of lipids packed within membranes of origin under study. For instance, folliculogenesis can be affected by the difference in the lipid composition and the different follicle sizes [46]. Annes et al. reported that the lipid composition of bovine oocytes from larger follicle sizes exhibits the highest lipid content—suggesting thar the follicular microenvironment of these follicle sizes support embryo development [46]. In different circumstances, spermatozoa is rich in phospholipids and cholesterol and shares membrane resemblance with cells. Although it has been disclosed that lipids are absent in seminal plasma, relatively small quantities of lipids in seminal plasma of bovine [47], boar [48], and human [49] were reported. Potentially, this condition affected how the fluorescent probe (i.e., CMG) selectively/specifically inserted their aliphatic tails into the EV’s lipid bilayer environment. Furthermore, the differences in the length and saturation of hydrocarbon (i.e., fatty acid) tails influence the rearrangement of phospholipid molecules to pack against one another [19]. This difference in the lipid composition of BFF and seminal plasma-derived EVs was also reflected in their respective ZP values. The above-mentioned cases along with vesicle heterogeneities can cause a great difference in labeling efficiencies between different EV sources.

It is worth noting that the technology employed in this study is constrained by its limitations. These limitations might include how the choice of the fluorescent probe relies on the question raised, the nature of the membrane system studied, and the measurement technique applied. NTA is capable of tracking and counting the number of detected particles under Brownian motion. However, it has a lower limit of detecting small particles less than 40 nm. This is why the NTA software needs to optimize a range of parameters, both in pre- and post-acquisition settings, to establish a much improved lower detection limit of smaller NPs. Though CMG particles are so bright and good scatterers, the *fl*-NTA instrument must be fitted with a high sensitivity camera along with choosing an appropriate fluorophore for measurements of labeled NPs in fluorescent mode. Additionally, the precise location of how the different fluorescent probes intercalated into the EV membrane is complex and poorly understood. Thus it needs proper understanding and design of custom fluorophores that bind specifically to EVs membrane. By doing so, it is possible to avoid any possible false-positive signals generated from dye particles, especially in in vivo EV internalization studies [50]. The aforementioned limitations need to be taken into consideration when choosing an efficient labeling method of EV membrane.

## 5. Conclusions

This study aimed to determine different conditions affecting CMG labeling of EVs derived from cell culture and biological fluids using fluorescence NTA. We optimized a range of parameters of fluorescence NTA for the detection of CMG labeled EVs. Higher concentrations of CMG led to the decrease of the detected *fl*-NP mean sizes originating from the JAr cells. This observed size shift infers the need to emphasize optimization of dye concentrations to preserve the size of EVs after labeling. The influence of different EV purification methods on the labeling ratios of *fl*-NPs originating from the JAr cell suggested the potential application of *fl*-NTA as an EV purification efficiency indicator. This study also evaluated the effects of incubation temperature on the physical characteristics of CMG labeled JAr EVs and confirmed that the labeled *fl*-NPs were bound by lipid membranes upon detergent treatment. Generally, the effect of CMG on the ZP of *fl*-NPs of JAr cell and biological origin was minimal. Further research should be undertaken to introduce *fl*-NTA with highly sensitive cameras that can handle NPs at lower brightness threshold levels. Besides, it might be possible to use dyes that are specific to a certain type of EVs, for example, exosomes and microvesicles. Currently, NTA is prone to error in discriminating EVs from non-EV particles and in measurements of their physical properties. This is why the adoption of fluorescence is necessary for EV research applications by NTA. Future studies may also explore the use of CMG dye and other lipophilic dye molecules to be fitted with other fluorescent labeling methods.

## Data Availability

Raw data for NTA are available in the Supplementary Materials. Analyzed data are either presented in the article or can be provided upon request.

## Supplementary Materials

The following are available online at https://www.mdpi.com/article/10.3390/membranes11100779/s1, Video S1: NTA data of JAr EVs only in 1× PBS solution, Video S2: Data of JAr EVs with CMG (100 ng/mL) in 1 × PBS solution measured in fluorescent mode of NTA, Video S3: Data of JAr EVs with CMG (100 ng/mL) in 1× PBS solution measured in scatter mode of NTA, Figure S1: TEM images of the JAr EVs purified with SEC. Scale bars of 500 nm are displayed within the figures, Figure S2: TEM images of the JAr EVs purified with TFF in combination with SEC. Scale bars of 500 nm are displayed within the figures.

## References

[1] Kalluri R., LeBleu V.S. (2020). The biology, function, and biomedical applications of exosomes. Science.

[2] Théry C., Amigorena S., Raposo G., Clayton A. (2006). Isolation and Characterization of Exosomes from Cell Culture Supernatants and Biological Fluids. Curr. Protoc. Cell Biol..

[3] Tkach M., Théry C. (2016). Communication by Extracellular Vesicles: Where We Are and Where We Need to Go. Cell.

[4] Yáñez-Mó M., Siljander P.R.-M., Andreu Z., Zavec A.B., Borràs F.E., Buzas E.I., Buzas K., Casal E., Cappello F., Carvalho J. (2015). Biological properties of extracellular vesicles and their physiological functions. J. Extracell. Vesicles.

[5] Van Niel G., D’Angelo G., Raposo G. (2018). Shedding light on the cell biology of extracellular vesicles. Nat. Rev. Mol. Cell Biol..

[6] Garofalo M., Villa A., Crescenti D., Marzagalli M., Kuryk L., Limonta P., Mazzaferro V., Ciana P. (2019). Heterologous and cross-species tropism of can-cer-derived extracellular vesicles. Theranostics.

[7] Van der Vlist E.J., Nolte-’t Hoen E.N.M., Stoorvogel W., Arkesteijn G.J.A., Wauben M.H.M. (2012). Fluorescent labeling of nano-sized vesicles released by cells and subsequent quantitative and qualitative analysis by high-resolution flow cytometry. Nat. Protoc..

[8] Tian Y., Manfei G., Gong M., Su G., Zhu S., Zhang W., Wang S., Li Z., Chen C., Li L. (2018). Protein Profiling and Sizing of Extracellular Vesicles from Colorectal Cancer Patients via Flow Cytometry. ACS Nano.

[9] Caponnetto F., Manini I., Skrap M., Palmai-Pallag T., Di Loreto C., Beltrami A.P., Cesselli D., Ferrari E. (2017). Size-dependent cellular uptake of exosomes. Nanomed. Nanotechnol. Biol. Med..

[10] Zhou X., Zhang J., Song Z., Lu S., Yu Y., Tian J., Li X., Guan F. (2020). ExoTracker: A low-pH-activatable fluorescent probe for labeling exosomes and monitoring endocytosis and trafficking. Chem. Commun..

[11] Marcu I.C., Eberhard N., Yerly A., Balmer V., Hemphill A., Mogel H., Gaschen V., Stoffel M.H., Bluteau J. (2020). Isolation of Human Small Extracellular Vesicles and Tracking of Their Uptake by Retinal Pigment Epithelial Cells In Vitro. Int. J. Mol. Sci..

[12] Tian T., Wang Y., Wang H., Zhu Z., Xiao Z. (2010). Visualizing of the cellular uptake and intracellular trafficking of exosomes by live-cell microscopy. J. Cell. Biochem..

[13] Kamerkar S., LeBleu V.S., Sugimoto H., Yang S., Ruivo C., Melo S., Lee J.J., Kalluri R. (2017). Exosomes facilitate therapeutic targeting of oncogenic KRAS in pancreatic cancer. Nat. Cell Biol..

[14] Kim S.M., Yang Y., Oh S.J., Hong Y., Seo M., Jang M. (2017). Cancer-derived exosomes as a delivery platform of CRISPR/Cas9 confer cancer cell tropism-dependent targeting. J. Control. Release.

[15] Meckes D.G., Shair K.H.Y., Marquitz A.R., Kung C.-P., Edwards R.H., Raab-Traub N. (2010). Human tumor virus utilizes exosomes for intercellular communication. Proc. Natl. Acad. Sci. USA.

[16] Wen S.W., Sceneay J., Lima L.G., Wong C.S., Becker M., Krumeich S., Lobb R., Castillo V., Ni Wong K., Ellis S. (2016). The Biodistribution and Immune Suppressive Effects of Breast Cancer–Derived Exosomes. Cancer Res..

[17] Li Y.-J., Wu J.-Y., Wang J.-M., Hu X.-B., Cai J.-X., Xiang D.-X. (2020). Gemcitabine loaded autologous exosomes for effective and safe chem-otherapy of pancreatic cancer. Acta Biomaterialia.

[18] Tang K., Zhang Y., Zhang H., Xu P., Liu J., Ma J., Lv M., Li D., Katirai F., Shen G.-X. (2012). Delivery of chemotherapeutic drugs in tumour cell-derived microparticles. Nat. Commun..

[19] Alberts B., Johnson A., Lewis J., Morgan D., Raff M., Roberts K., Walter P., Wilson J., Hunt T. (2002). Molecular Biology of the Cell.

[20] Sengupta S., Rothenberg K.E., Li H., Hoffman B.D., Bursac N. (2019). Altering integrin engagement regulates membrane localization of K ir 2.1 channels. J. Cell Sci..

[21] Bianchi F., Pereno V., George J.H., Thompson M.S., Ye H. (2019). Membrane Mechanical Properties Regulate the Effect of Strain on Spontaneous Electrophysiology in Human iPSC-Derived Neurons. Neuroscience.

[22] Vogt E.-J., Tokuhiro K., Guo M., Dale R., Yang G., Shin S.-W., Movilla M.J., Shroff H., Dean J. (2019). Anchoring cortical granules in the cortex ensures trafficking to the plasma membrane for post-fertilization exocytosis. Nat. Commun..

[23] Mathieu M., Martin-Jaular L., Lavieu G., Théry C. (2019). Specificities of secretion and uptake of exosomes and other extracellular ves-icles for cell-to-cell communication. Nat. Cell Biol..

[24] Dragovic R.A., Gardiner C., Brooks A.S., Tannetta D.S., Ferguson D., Hole P., Carr B., Redman C.W., Harris A., Dobson P.J. (2011). Sizing and phenotyping of cellular vesicles using Nanoparticle Tracking Analysis. Nanomed. Nanotechnol. Biol. Med..

[25] Bachurski D., Schuldner M., Nguyen P.-H., Malz A., Reiners K.S., Grenzi P.C., Schauss A.C., Hansen H.P., Hallek M., Von Strandmann E.P. (2019). Extracellular vesicle measurements with na-noparticle tracking analysis—An accuracy and repeatability comparison between NanoSight NS300 and ZetaView. J. Extracell. Vesicles.

[26] Dissanayake K., Nõmm M., Lättekivi F., Ressaissi Y., Godakumara K., Lavrits A., Midekessa G., Viil J., Bæk R., Jørgensen M.M. (2020). Individually cultured bovine embryos produce extracellular vesicles that have the potential to be used as non-invasive embryo quality markers. Theriogenology.

[27] Giebel B., Helmbrecht C. (2017). Methods to Analyze EVs. Methods Mol. Biol..

[28] Dissanayake K., Midekessa G., Lättekivi F., Fazeli A., Brevini T.A., Fazeli A., Turksen K. (2021). Measurement of the Size and Concentration and Zeta Potential of Extracellular Vesicles Using Nanoparticle Tracking Analyzer. Next Generation Culture Platforms for Reliable In Vitro Models. Methods in Molecular Biology.

[29] Carnell-Morris P., Tannetta D., Siupa A., Hole P., Dragovic R. (2017). Analysis of Extracellular Vesicles Using Fluorescence Nanoparticle Tracking Analysis. Membrane Trafficking.

[30] CellMaskTM Green Plasma Membrane Stain. https://www.thermofisher.com/order/catalog/product/C37608.

[31] Es-Haghi M., Godakumara K., Häling A., Lättekivi F., Lavrits A., Viil J., Andronowska A., Nafee T., James V., Jaakma U. (2019). Specific trophoblast transcripts transferred by ex-tracellular vesicles affect gene expression in endometrial epithelial cells and may have a role in embryo-maternal crosstalk. Cell Commun. Signal..

[32] Van Deun J., Mestdagh P., Agostinis P., Akay Ö., Anand S., Anckaert J., Martinez Z.A., Baetens T., Beghein E., EV-TRACK Consortium (2017). EV-TRACK: Transparent reporting and centralizing knowledge in extracellular vesicle research. Nat. Methods.

[33] Kornilov R., Puhka M., Mannerström B., Hiidenmaa H., Peltoniemi H., Siljander P., Seppänen-Kaijansinkko R., Kaur S. (2018). Efficient ultrafiltration-based protocol to deplete extracellular vesicles from fetal bovine serum. J. Extracell. Vesicles.

[34] Théry C., Witwer K.W., Aikawa E., Alcaraz M.J., Anderson J.D., Andriantsitohaina R., Antoniou A., Arab T., Archer F., Atkin-Smith G.K. (2018). Minimal information for studies of extracellular vesicles 2018 (MISEV2018): A position statement of the International Society for Extracellular Vesicles and update of the MISEV2014 guidelines. J. Extracell. Vesicles.

[35] Midekessa G., Godakumara K., Ord J., Viil J., Lättekivi F., Dissanayake K., Kopanchuk S., Rinken A., Andronowska A., Bhattacharjee S. (2020). Zeta Potential of Extracellular Vesicles: Toward Understanding the Attributes that Determine Colloidal Stability. ACS Omega.

[36] Hasan M.M., Viil J., Lättekivi F., Ord J., Reshi Q., Jääger K., Velthut-Meikas A., Andronowska A., Jaakma Ü., Salumets A. (2020). Bovine Follicular Fluid and Extracellular Vesicles Derived from Follicular Fluid Alter the Bovine Oviductal Epithelial Cells Transcriptome. Int. J. Mol. Sci..

[37] Gardiner C., Ferreira Y.J., Dragovic R.A., Redman C.W., Sargent I.L. (2013). Extracellular vesicle sizing and enumeration by nanoparticle tracking analysis. J. Extracell. Vesicles.

[38] Shimomura T., Seino R., Umezaki K., Shimoda A., Ezoe T., Ishiyama M., Akiyoshi K. (2021). New Lipophilic Fluorescent Dyes for Labeling Extracellular Vesicles: Characterization and Monitoring of Cellular Uptake of Exosomes. Bioconjugate Chem..

[39] Dehghani M., Gulvin S.M., Flax J., Gaborski T.R. (2020). Systematic Evaluation of PKH Labelling on Extracellular Vesicle Size by Na-noparticle Tracking Analysis. Sci. Rep..

[40] Auría-Soro C., Nesma T., Juanes-Velasco P., Landeira-Viñuela A., Fidalgo-Gomez H., Acebes-Fernandez V., Gongora R., Parra M.J.A., Manzano-Roman R., Fuentes M. (2019). Interactions of Nanoparticles and Biosystems: Microenvironment of Nanoparticles and Biomolecules in Nanomedicine. Nanomaterials.

[41] Jensen E.C. (2012). Use of Fluorescent Probes: Their Effect on Cell Biology and Limitations. Anat. Rec. Adv. Integr. Anat. Evol. Biol..

[42] Tertel T., Bremer M., Maire C., Lamszus K., Peine S., Jawad R., Andaloussi S.E., Giebel B., Ricklefs F.L., Görgens A. (2020). High-Resolution Imaging Flow Cytometry Reveals Impact of Incubation Temperature on Labeling of Extracellular Vesicles with Antibodies. Cytom. Part A.

[43] Osteikoetxea X., Sódar B., Németh A., Szabó-Taylor K., Pálóczi K., Vukman K.V., Tamasi V., Balogh A., Kittel A., Pallinger E. (2015). Differential detergent sensitivity of extra-cellular vesicle subpopulations. Organ. Biomol. Chem..

[44] Musante L., Saraswat M., Duriez E., Byrne B., Ravida A., Domon B., Holthofer H. (2012). Biochemical and Physical Characterisation of Urinary Nanovesicles following CHAPS Treatment. PLoS ONE.

[45] György B., Módos K., Pállinger É., Pálóczi K., Pásztói M., Misják P., Deli M., Sipos Á., Szalai A., Voszka I. (2011). Detection and isolation of cell-derived microparticles are compromised by protein complexes resulting from shared biophysical parameters. Blood.

[46] Annes K., Müller D.B., Vilela J.A.P., Valente R.S., Caetano D.P., Cibin F.W.S., Milazzotto M., Mesquita F.S., Belaz K.R.A., Eberlin M.N. (2019). Influence of follicle size on bovine oocyte lipid composition, follicular metabolic and stress markers, embryo development and blastocyst lipid content. Reprod. Fertil. Dev..

[47] Komarek R., Pickett B., Lanz R., Jensen R. (1964). Lipid Composition of Bovine Spermatozoa and Seminal Plasma. J. Dairy Sci..

[48] Am-In N., Kirkwood R., Techakumphu M., Tantasuparuk W. (2011). Lipid profiles of sperm and seminal plasma from boars having normal or low sperm motility. Theriogenology.

[49] Vignon F., Koll-back M.H., Clavert A., Cranz C. (1989). Lipid Composition of Human Seminal Plasma. Arch. Androl..

[50] Pužar Dominkuš P., Stenovec M., Sitar S., Lasič E., Zorec R., Plemenitaš A., Žagar E., Kreft M., Lenassi M. (2018). PKH26 labeling of extracellular vesicles: Characterization and cellular internalization of contaminating PKH26 nanoparticles. Biochim. Biophys. Acta (BBA)-Biomembr..

