# Supplementary material for: Characterization of Extracellular Vesicles Labelled with a Lipophilic Dye Using Fluorescence Nanoparticle Tracking Analysis
Source: Membranes (Basel). 2021 Oct 12;11(10):779. doi: 10.3390/membranes11100779 (PMC8539200; doi:10.3390/membranes11100779)
Supplement: Supplementary file 1 [file membranes-11-00779-s001.zip › membranes-1393973-supplementary.pdf]

membranes

Article

Characterization of Extracellular Vesicles Labeled with a Lipophilic Dye Using Fluorescence Nanoparticle Tracking Analysis

Getnet Midekessa [1,2], Kasun Godakumara [1,2], Keerthie Dissanayake [1,2,3], Mohammad Mehedi Hasan [1,2], Qurat Ul Ain Reshi [1,2], Toonika Rinken [4] and Alireza Fazeli [1,2,5,*]

1 Institute of Veterinary Medicine and Animal Sciences, Estonian University of Life Sciences, Kreutzwaldi 62, 51006 Tartu, Estonia; getnet.balcha@ut.ee (G.M.); kasun.godagedara@ut.ee (K.G.); keerthie.dissanayake@ut.ee (K.D.); mehedi.hasan@ut.ee (M.M.H.); qurat.reshi@ut.ee (Q.U.A.R.)
2 Department of Pathophysiology, Institute of Biomedicine and Translational Medicine, University of Tartu, Ravila St. 14b, 50411 Tartu, Estonia
3 Department of Anatomy, Faculty of Medicine, University of Peradeniya, Peradeniya 20400, Sri Lanka
4 Institute of Chemistry, University of Tartu, Ravila St. 14a, 50411 Tartu, Estonia; toonika.rinken@ut.ee
5 Academic Unit of Reproductive and Developmental Medicine, Department of Oncology and Metabolism, Medical School, University of Sheffield, Sheffield S10 2SF, UK
* Correspondence: alireza.fazeli@ut.ee; Tel.: +372-737-4425

Citation: Midekessa, G.; Godakumara, K.; Dissanayake, K.; Hasan, M.M.; Reshi, Q.U.A.R.; Rinken, T.; Fazeli, A. Characterization of Extracellular Vesicles Labeled with a Lipophilic Dye Using Fluorescence Nanoparticle Tracking Analysis. *Membranes* **2021**, *11*, 779. https://doi.org/10.3390/membranes11100779

Academic Editor: Shiro Suetsugu

Received: 8 September 2021
Accepted: 6 October 2021
Published: 12 October 2021

Publisher's Note: MDPI stays neutral with regard to jurisdictional claims in published maps and institutional affiliations.

Copyright: © 2021 by the authors. Licensee MDPI, Basel, Switzerland. This article is an open access article distributed under the terms and conditions of the Creative Commons Attribution (CC BY) license (http://creativecommons.org/licenses/by/4.0/).

1. Supplementary information

Video S1: NTA data of JAr EVs only in 1× PBS solution (AVI).

Video S2: Data of JAr EVs with CMG (100 ng/mL) in 1× PBS solution measured at the fluorescent mode of NTA (AVI).

Video S3: Data of JAr EVs with CMG (100 ng/mL) in 1× PBS solution measured at scatter mode of NTA (AVI)

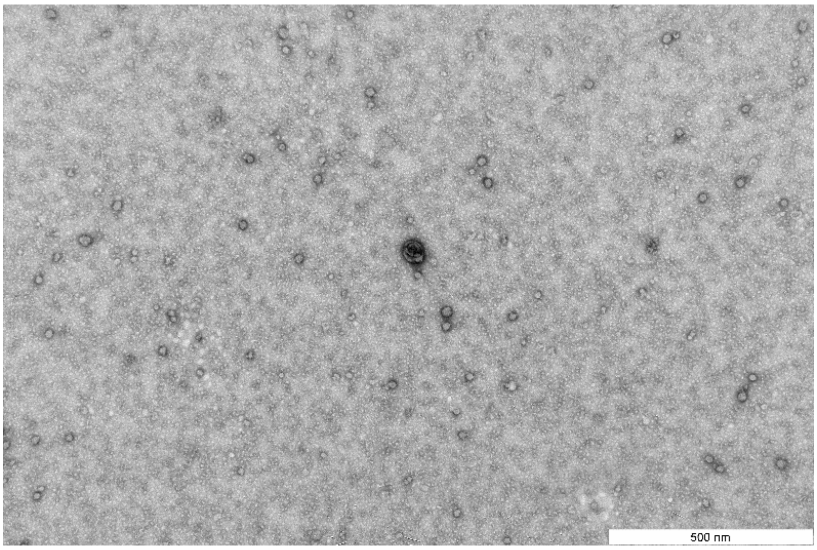

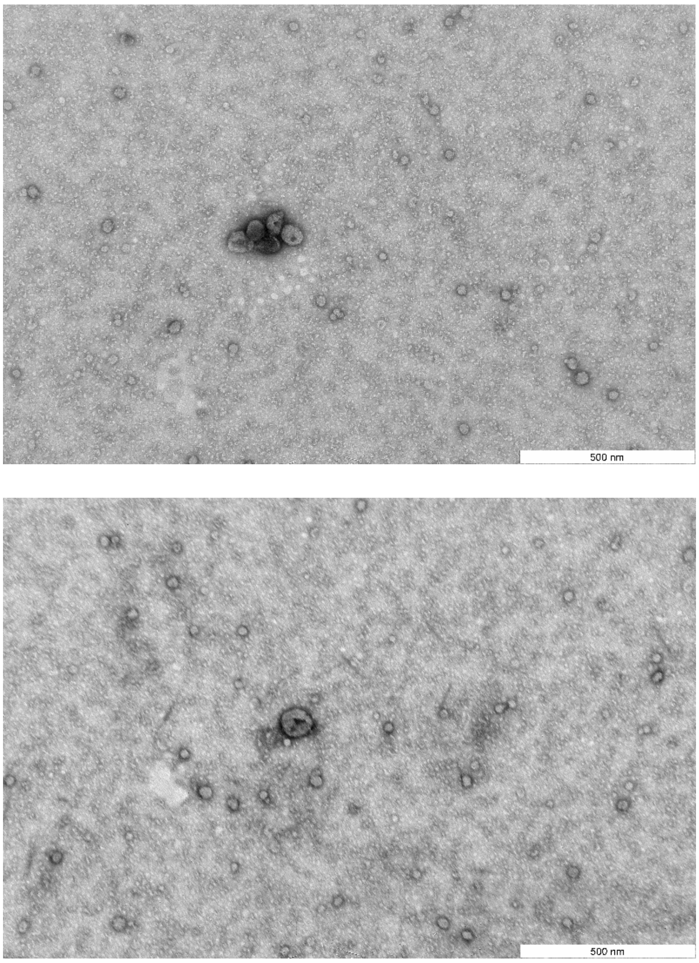

Figure S1. TEM images of the JAr EVs purified with SEC. Scale bars of 500 nm are displayed within the figures.

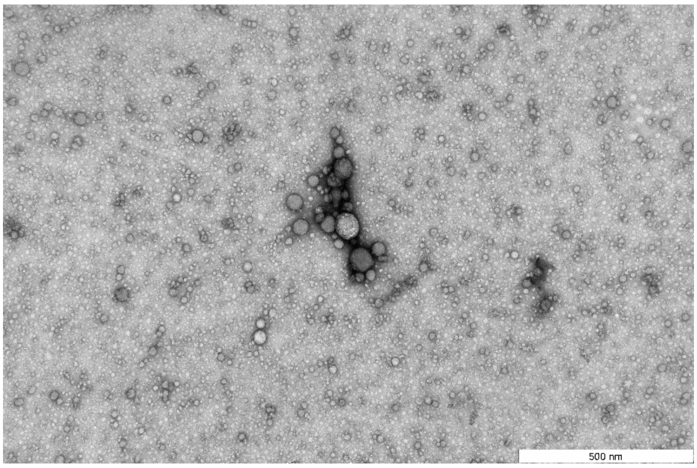

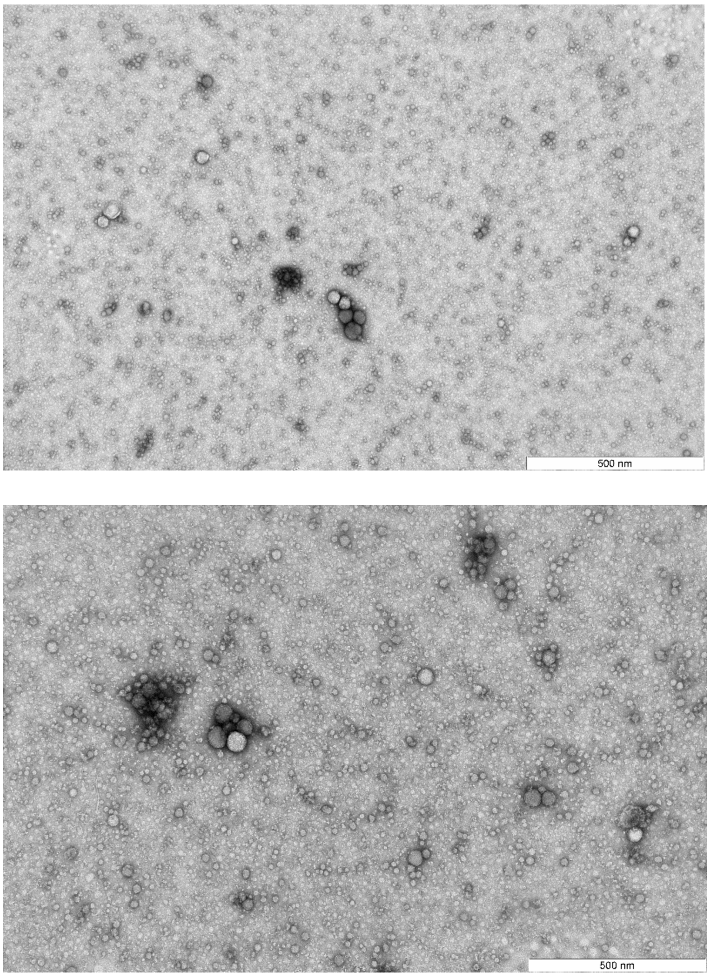

Figure S2. TEM images of the JAr EVs purified with TFF in combination of SEC. Scale bars of 500 nm are displayed within the figures.
